# The microbiome and lung cancer: microbial effects on host immune responses and treatment outcomes

**DOI:** 10.3389/frmbi.2025.1606551

**Published:** 2025-09-01

**Authors:** Alexis Bailey, Kerstin K. Leuther, Lary A. Robinson

**Affiliations:** ^1^ Department of Thoracic Oncology, Moffitt Cancer Center, Tampa, FL, United States; ^2^ Department of Clinical Research, University of Jamestown, Jamestown, ND, United States

**Keywords:** non-small cell lung cancer, gut microbiome, immune response, lung microbiome, inflammation, probiotics, immunotherapy, gut-lung axis

## Abstract

The human microbiome plays a critical role in shaping physiological processes, immune system function, metabolism, and disease development. Recent research has highlighted the microbiome’s profound cancer impact, particularly on lung cancer. This review explores how microbial communities in lung and gut influence tumor progression, immune responses, and treatment outcomes as well as describing the interactions between the microbiome and the host immune system in modulating the efficacy of cancer therapies. Emerging evidence from preclinical and clinical studies investigating the role of the lung and gut microbiome in lung cancer focus on alterations in the microbiota that influence the tumor microenvironment, modulate immune responses, and potentially enhance/hinder treatment effectiveness such as chemotherapy, targeted therapies, and immunotherapy. Microbial diversity plays a significant role in immune regulation, and specific microbial species may activate/suppress immune cells such as T-cells, dendritic cells, and macrophages. Furthermore, this review examines the therapeutic implications of microbiome modulation, including the use of probiotics, antibiotics, and fecal microbiota transplantation in enhancing cancer therapies. Alterations in the lung and gut microbiome and their interaction in the recently described gut-lung axis with its bidirectional communication significantly influence the tumor microenvironment and systemic immune responses. These findings suggest that microbial diversity can regulate immune functions, with specific species capable of activating or suppressing immune cell activity. Furthermore, microbiome-targeted interventions show potential in improving the effectiveness of treatments including chemotherapy, targeted therapies, and immunotherapy, underscoring the importance of the microbiome as a key factor in lung cancer pathogenesis and treatment.

## 1 Introduction

Microorganisms within the human body are present in exponentially greater numbers compared to the total number of human cells (Sender et al., 2016). In fact, the most recent estimate is that there are 3 x 10^13^ human cells in the average 70 kg male human body and an estimated 3.8 x 10^13^ symbiotically living microorganisms (1.3 ratio bacteria/human cells) that harmoniously interact with their host cells (Gilbert et al., 2018). Numerous microbial species colonize distinct niches and organs within the ecosystem of the human body, with the colon containing the largest number. Although the total bacterial mass is only 0.2 kg, their density and biological processes are susceptible to fluctuations due to exogenous and endogenous factors, including age, sex, and body size (Sender et al., 2016). The variations of these factors has led investigators to investigate the impact of the microbiome on human health and diseases (Lynch and Pedersen, 2016).

Eubiosis, or a “healthy” microbiome, has not been well characterized by the scientific community since it is difficult to distinguish eubiosis from dysbiosis, or an “unhealthy” microbiome. However, distinct dysbiotic microbial signatures are associated with disease and are being uncovered using techniques such as high throughput sequencing, that allow surveying the human microbiome. High throughput sequencing has led to discoveries of how these commensal microbiota play a role in modulating progression of diseases, including cancer, and the data suggest that specific pathogens or shifts in microbiota communities can contribute to carcinogenesis (Allen and Sears, 2019).

Genetic mutations appear to be the primary drivers for tumor initiation and progression, in addition to secondary risk factors including age, diet, environmental exposures, and obesity. Additionally, advances in microbiome research have shown that microorganisms affect the progression in tumor mutations (Kadosh et al., 2020). Evidence suggests that these host microbial signatures may aid in the prediction of the initial stage of tumor formation (Poore et al., 2020), correlate with the survival rate in a specific cancer (Riquelme et al., 2019), and even influence the effect of immunotherapeutic responses and toxicity (Gopalakrishnan et al., 2018).

Lung cancer is the third most common U.S. cancer following breast and prostate cancer, but it is by far the leading cause of cancer deaths in both male and females with over 125,000 U.S. deaths in 2024 (Siegel et al., 2024)–more than the next three cancers combined. Lung cancer is also the most common cancer worldwide leading to the most deaths, with over 1.8 million deaths worldwide (Bray et al., 2024; Siegel et al., 2024). After extensive studies at the molecular level and characterization of both the genomic landscape and genetic mutations, lung cancer appears to be highly heterogeneous. Based on genetic research, numerous advances in targeted therapies as well as immunotherapies that harness the immune system have been realized. Despite these efforts, overall survival rates for lung cancer remain extremely low with approximately a 25% five-year survival rate for all stages combined, resulting in the highest cancer-related death rate of any cancer (Siegel et al., 2024).

Many risk factors impact the development of lung cancer, with cigarette smoking being the primary factor contributing to lung carcinogenesis (Le Noci et al., 2018). Although other environmental risk factors have been identified, the mechanism explaining how these factors contribute to carcinogenesis is poorly understood. Although the lungs were originally thought to be sterile, the lungs have the largest surface area in the human body providing gas exchange from the outside world, and the belief of the lung as a sterile organ has been disproven by numerous studies (Dickson et al., 2016). Since the lungs are exposed to a multitude of microorganisms, we then question whether the now-recognized diverse lung microbiome is associated with lung cancer (Dickson and Huffnagle, 2015).

The role of the lung and other organ microbiomes in cancer evolution has been extensively studied demonstrating that cancer cells themselves are affected by the microbiome. Additionally, the microbiome can also regulate cancer immunosurveillance. This review will explore the microbiome in relationship to lung cancer development and its effects on immune interactions with this disease, as well as discuss how this immunomodulation can impact tumor carcinogenesis in lungs and immunotherapy toxicities.

## 2 Cancer and the immune system

In the past, limitations in technology prevented scientists from exploring how the immune system may affect growth suppression in cancers. Only during the last decade were researchers able to investigate Paul Ehrlich’s hypothesis that the immune system can suppress carcinoma growth (Ehrlich, 1908). Since then, numerous advances in tumor immunology and the understanding of cancer immunoediting as related to immunotherapy have transformed the treatment landscape (Dunn et al., 2004). Cancer immunoediting refers to the important interaction between host immune cells and the cancer that occurs during tumor growth and while receiving immunotherapy. Immunoediting can both constrain and promote tumor growth and has been categorized by O’Donnell et al. (2019) into three phases: elimination, equilibrium, and escape (**Table 1**).

**Table 1:** Immunoediting phases (O’Donnell et al., 2019).

Phases	Cells involved	Effect
Elimination (Immunosurveillance)	Transformed	Transformed cells are destroyed by the innate and adaptive immune system.
Equilibrium	Tumor clones	Clones surviving elimination may progress to where tumor growth is limited and even stalled.
Escape	Tumor clones	Genetic instability may lead to reduced immunogenicity with evasion of immune destruction.

In the *elimination* phase, also known as immunosurveillance, transformed cells are destroyed by a functional immune system. However, tumors have developed mechanisms to avoid the elimination phase, which is carried out by adaptive immune cells such as natural killer cells. Cytotoxicity is mediated by production of effectors, like IFN-*γ*, through the advancement of tumor intrinsic pathways (Wellenstein and de Visser, 2018), by interacting with immune cells, and by interacting with the tumor stroma within the tumor microenvironment (Chen et al., 2015). Sporadically, some transformed cell clones survive elimination and enter the *equilibrium* phase in which clones may progress to limited or even stalled growth. However immune cells, and their metabolic products including cytokines, can paradoxically become tumor-promoting through inflammation within the tumor microenvironment during tumor progression (Chen et al., 2015). The third phase is *escape* where immune-modified tumors with reduced immunogenicity grow and become clinically apparent. Nevertheless, cancer immunoediting presents several molecular pathways that could be targeted to overcome immunoresistance and cease tumor growth (Zhang and Zhang, 2020).

The recent discoveries of the interplay between the immune system and cancer have led to therapies directed at stimulating anti-tumor immune responses with promising results in clinical trials. A few examples include the use of CAR-T cells (June et al., 2018), immune checkpoint inhibitors (Wei et al., 2018), and CpG oligonucleotides (Adamus and Kortylewski, 2018). Although it is important to understand the effects of the immune system and carcinogenesis in designing approaches to immunotherapy, it’s also important to appreciate how the immune system affects treatment outcomes for other anti-tumor therapies such as chemotherapy (Bracci et al., 2014) and radiotherapy (Walle et al., 2018).

## 3 Microbiome interaction with immune cells during cancer initiation and progression

Microbial communities, even individual microorganisms, can transform the relationship between the immune system and cancer by altering the innate and adaptive immune system within the host (**Table 2**). Microorganisms can increase the initiation and progression of cancer while also modulating cancer immunosurveillance.

**Table 2:** Methods of modulation of innate and adaptive immune system response by microbiome.

Cell types	Purpose	Effects
Innate cells		
Natural killer T cells (NKT cells)	Cytotoxin immunosurveillance	Can be downregulated by commensal bacteria, vancomycin can reverse this negative effect by removing the gram-positive bacteria.
Adaptive cells		
Host CD4+ and CD8+ T cells	Secrete IFN-γ	Associated with positive clinical outcomes depending on the microbiome that is present.
Tumor associated macrophages	Cytokines and chemokines	Induced by dysbiosis producing cytokines and chemokines within the tumor microenvironment that suppress cytotoxicity of T cells and promote tumor growth and even metastases.
Cells inducing tumor-inhibiting IL-9	Restore Il-9	Fecal microbiota transplant can restore microbiota and restore Il-9 production to decrease tumor growth.
Gamma-delta T cells (γδ T cells)	Promote inflammatory response	Promote inflammatory response filling the gap between the adaptive and innate immune system. γδ T cells are able to recognize diverse antigens and still create an immune response against that antigen.
Follicular T helper cells	Immunogenic agents or as tolerogenic agents	Found in tumor draining lymph nodes and depending on the microbiome they act as immunogenic agents or as tolerogenic agents.

Microbial communities can negatively and positively impact the innate and adaptive immune cell systems and can either upregulate or deregulate key immune cells that aid in cancer detection or depletion.

### 3.1 Innate immune system

Within the innate immune system, natural killer T cells (NKT cells) are involved in the immunosurveillance of cancer due to their cytotoxic properties (Nair and Dhodapkar, 2017). When commensal gut bacteria downregulate the recruitment and concentration of CXCR6+ NKT cells in liver tumors, tumor progression continues and increases (Ma et al., 2018). In a murine liver cancer model, Ma et al. (2018) found that the microbiome could be modulated by removing gram positive bacteria with the use of vancomycin to reverse the effects of these microorganisms and enable NKT cells to repopulate liver tumors to mediate an anti-tumor immune response.

### 3.2 Adaptive immune system

The adaptive immune system hosts CD4+ and CD8+ T cells that secrete IFN-*γ*, and these cells have been associated with positive clinical outcomes in patients with pancreatic ductal adenocarcinomas (Sethi et al., 2018).

#### 3.2.1 CD4+ and CD8+ T cells

The gut microbiome can modulate the adaptive immune response and potentially decrease the number of CD4+ and CD8+ T cells that are recruited to the cancer site. Sethi et al. (2018) showed that oral antibiotic-mediated depletion of portions of the gut microbiome in a mouse model with pancreatic tumors increased CD8+ secreting IFN-*γ* T cells, resulting in reduced tumor burden. Conversely microorganisms interacting with CD4+ and CD8+ T cells have also been associated with improved responses to cancer immunotherapies and chemotherapies.

Daillère et al. (2016) report that the species *Enterococcus hirae* translocated to the secondary lymphoid organs from the small intestine during cyclophosphamide treatment of sarcoma tumors. As a result, this microbe increased the intratumoral CD8+ to T_reg_ ratio. Accumulation of another microbe species, *Barnesiella intestinihominis*, promoted recruitment of *γδ*T cells that produce IFN-*γ* within the sarcoma lesion. This discovery was corroborated by Vétizou et al. (2015), observing that the species *Bacteroides fragilis* responsive T cells modulate and enhance anti-CTLA4 immunotherapy efficacy in sarcomas. In non-small cell lung cancer, Routy et al. (2018) found that relative abundance of the species *Akkermansia muciniphila* induced CD4+ T cell production restoring the efficacy of anti-PD-1 therapy.

Typically, the immune cells that first encounter microorganisms are dendritic cells through pattern recognition receptors (PRRs) and they act as an antigen within the adaptive immune system. Paulos et al. (2007) found in a mouse melanoma model that lymphodepletion by total body irradiation increased tumor-specific CD8 T cells and increased cytokine levels via TLR4-mediated signaling. During radiotherapy in this model, gut microorganisms activate dendritic cells, and initiate an anti-tumor effect by recruiting CD8 T cells (Paulos et al., 2007). Uribe-Herranz et al. (2020) showed that gut microorganisms can modulate anti-tumor response by the presentation of antigens on dendritic cells and decrease CD8 T cells in mice models with melanoma and lung cancer responding to radiotherapy. Additionally, these investigators were able to demonstrate modulation of the gut microbiome with the use of vancomycin to increase IL-12 secretion via CD8+ dendritic cells which improved the T cell response in lung cancer and cervical cancer (Uribe-Herranz et al., 2018). Increased IL-12 and IL-1 secretion within tumor tissue activates a cytotoxic T cell response within the gut microbiome. In multiple tumor models, an increase in cytokine secretion has been shown to mediate the immunomodulatory effects of chemotherapy and immunotherapy (Vétizou et al., 2015).

#### 3.2.2 Tumor associated macrophages

Tumor associated macrophages (TAMs) produce cytokines and chemokines within the tumor environment that can suppress cytotoxic T cells, thus helping to promote tumor growth and even foster tumor metastasis (Pathria et al., 2019). Dysbiosis can contribute to TAM induction through TLR4 signaling which creates an environment that promotes tumor growth due to immunosuppression (Li et al., 2019). Specific microbial species can accelerate colorectal cancer progression, such as *Fusobacterium nucleatum*, through modulation of the innate immune system inducing TAMs within the tumor microenvironment, which results in a suppressed T cell response (Kostic et al., 2013).

#### 3.2.3 Microbial induction of tumor-inhibiting IL-9

Within the colonic lamina propria, the gut microbiome is essential for producing IL-9 to induce Th9 (CD4+ T Helper 9 Cells) and Tc9 (CD8+ Cytotoxic T 9 Cells) cells. Almeida et al. (2020) demonstrated in germ-free (GF) mice a decreased expression of IL-4 and TGF-ß which resulted in a reduction of Th9 cells that led to an increase in melanoma tumor growth subcutaneously. In the same study, a fecal microbiota transplant into these GF mice from conventional mice restored IL-9 production and decreased tumor growth (Almeida et al., 2020). Miao et al. (2017) showed evidence of microbial modulation in squamous cell carcinoma (SCC) in a mouse model where Th9 cells exposed to Staphylococcal enterotoxin B began to expand with SCC antigens which resulted in an increase in cancer cell apoptosis. Staphylococcal enterotoxin B increased levels of STAT5, HDAC1, and PU.1 significantly in CD4+ T cells which led to an increase in the production of Il-9 secretion (Miao et al., 2017).

#### 3.2.4 Gamma-delta T cells (γδ T cells)

Gamma-delta T cells (*γδ* T cells) play a key role in the immune response by promoting the inflammatory responses of lymphoid and myeloid lineages. They are considered to bridge the gap between the adaptive and innate immune systems. These cells can express T-cell receptors but are unique in that they do not exhibit antigen recognition through MHC molecules. As a result, *γδ* T cells are able to recognize diverse antigens and still promote an immune response against that antigen (Holtmeier and Kabelitz, 2005). In a germ-free or antibiotic-treated mouse lung adenocarcinoma model, the addition of commensal bacteria led to the production of IL-17, which in turn generated γδ T cells in mice harboring genetically modified KRAS and P53 mutations that conferred protection. Evidently, the immunosuppressed tumor microenvironment and tumor cell proliferation were facilitated by the presence of commensal bacteria (Cheng et al., 2014; Jin C. et al., 2019). These investigators then were able to restore the anti-cancer response by removing the microbiome, thus upregulating the production of IFN- *γ* in *γδ* T cells. Distinct taxa of bacteria were identified within cancer-bearing lungs such as *Herbaspirillum* and *Sphingomonadaceae*, in comparison to healthy lungs containing taxa *Aggregatibacter* and *Lactobacilli* (Jin C. et al., 2019). The immune system-microbiome interaction is highly specific since effects in one area of the host may have completely different outcomes in another area of the body. Pulmonary melanoma metastasis models paradoxically had an opposite outcome when compared to the previous lung adenocarcinoma study as administration of antibiotics led to *γδ* T cell and IL-17 production resulting in accelerated pulmonary metastases (Cheng et al., 2014).

#### 3.2.5 Follicular T helper cells

Follicular T helper cells (T_FH_) cells are most abundant within the mucosal lymphoid tissue and can also be found concentrated in tumor draining lymph nodes. Follicular T helper cells are also a part of the adaptive immune system and are involved in immune system-to-cancer interactions (Roberti et al., 2020). Intestinal epithelial cell apoptosis was shown to induce T_FH_ which resulted in a reduction of tumor growth (Roberti et al., 2020). Roberti et al. (2020) determined that this effect was dependent upon the commensal ileal microbiome with some of these bacteria acting as immunogenic agents and others as tolerogenic agents.

The body of evidence just described reports that microbial communities shape many of the innate and adaptive immune responses and can regulate the production of some immune cells. Immune cells are tailored based upon the presence of certain microbiota within the tumor or the tumor microenvironment and can change how the immune system will respond to tumor progression or tumor immunosurveillance and regression. Most importantly, microbial communities can negatively and positively impact the innate and adaptive immune systems and can either upregulate or downregulate key immune cells that aid in cancer detection or depletion.

## 4 Tumor-promoting microbial inflammation

The relationship between inflammation and cancer pathogenesis is readily suggested by correlating the increased incidence of cancer in people with chronic inflammation due to underlying diseases. Chronic inflammation is a side effect of many diseases and conditions such as irritable bowel disease, pancreatitis, ulcerative colitis, obesity, and others which can then foster tumor initiation and tumor growth (Greten and Grivennikov, 2019). Accepted causes of chronic lung inflammation are listed in **Table 3**.

**Table 3:** Causes of chronic lung inflammation.

Causes	Effects
Patient factors	
Chronic lung diseases	Increased the level of inflammatory cytokines such as IL-1β, IL-6
Chronic lung infections including post-obstructive pneumonia	Increased the level of inflammatory cytokines such as IL-1β, IL-6
Immunocompromised state	Impaired immune function
Autoimmune diseases	Impaired immune function
Certain medications	Impaired immune function
Microbiome	
Low lung microbiome alpha diversity	Promotes a favorable inflammatory response due to high chemokine expression resulting in enhanced T cell infiltration
Microaspiration of oral microbiota	Increase local immune tone with upregulation of IL1, IL6, and ERK/MARK
Adverse lung microbiota composition (dysbiosis)	Impaired immune function
External causes	
Cigarette smoke	Inhaled foreign antigens cause increased levels of inflammatory cytokines such as IL-1β, IL-6
Occupational hazards	Inhaled foreign antigens cause increased levels of inflammatory cytokines such as IL-1β, IL-6
Air pollution	Inhaled foreign antigens cause increased levels of inflammatory cytokines such as IL-1β, IL-6

Comparable to chronic inflammation, microbial-induced inflammation is also postulated to be tumorigenic. For example, the species *Heliobacter pylori* gastritis may lead to the development of gastric cancer (Lamb and Chen, 2013) and the parasite *Schistosoma haematobium* infection may result in bladder cancer (Mostafa et al., 1999), with both believed due to chronic inflammation. Microorganisms can also modulate cancer development and even stimulate progression such as that seen in colon cancer. For colorectal tumors, *Fusobacterium* species have been shown to promote immunosuppression that inhibits NK cell cytotoxicity (Gur et al., 2015), T-cell activity (Kaplan et al., 2010), increase suppressor cells (Kostic et al., 2013), increase tumor macrophages (Park et al., 2017), and suppress tumor infiltrating lymphocytes (Hamada et al., 2018).

### 4.1 Gut microbiome influence on inflammation

Prior to affecting established tumors, the gut microbiome has been shown to contribute to tumorigenic inflammation in precursor lesions as well. Gaiser et al. (2019) detected an increase of oral microorganisms in tissue specimens that were resected from the human pancreas. The microenvironment included the species *Fusarium nucleatum* which was significantly denser in patients with invasive pancreatic cancer compared to patients that did not have pancreatic cancer. To further support this theory, Gaiser demonstrated elevated levels of intra-cystic IL-1*β* in the invasive cancer group which positively correlates with 16S bacterial DNA. This finding suggests that *F. nucleatum* present within the malignant lesions may have increased the level of inflammatory cytokines recruited to the area creating a tumorigenic environment during the development of the initial tumor (Gaiser et al., 2019).

In contrast to inflammation contributing to cancer, recent studies have also shown positive potential *anti-tumorigenic* microbial inflammation associated with other microbial species, as opposed to the tumorigenic inflammation caused by *Fusobacterium* in colorectal cancer. Bacterial families such as *Lachnospiraceae* and *Ruminococcaceae* found in biopsies from colon cancer promote a favorable inflammatory response due to high chemokine expression resulting in enhanced T cell infiltration (Cremonesi et al., 2018). Therefore, the gut microbiome exists in a balance where certain microbial species can either positively induce tumor formation or negatively impact tumor initiation, depending on which microbial communities predominate.

Not only do the gut microbiota contribute to tumorigenic inflammation but also they play a key role in common benign gastrointestinal disease such as inflammatory bowel disease, irritable bowel syndrome and celiac disease (Ullah et al., 2025). Autoimmune disorders, metabolic disorders including diabetes, cardiovascular disease, chronic kidney disease and even neurodegenerative diseases are linked to an adverse gut microbiota, likely due to its impairment of immune function (Ullah et al., 2024). The gut microbiota also interact with the central nervous system in the gut-brain axis, a bidirectional system with signaling pathways including chemical neurotrophic factors as well as endocrine and immunologic systems (Ullah et al., 2023). Evidence strongly suggests that gut dysbiosis affects brain-related activities linked to the development and progression of neurodegenerative diseases such as Alzheimer’s disease, Parkinson’s disease, multiple sclerosis and autism spectrum disorder (Ullah et al., 2023).

### 4.2 Lung microbiome inflammation in lung cancer

The lungs are routinely presented with environmental microorganisms that colonize the airways and parenchyma. Originally it was thought that localized flora in the lungs contributed to the initiation of regulatory T cells to reduce excessive inflammatory responses due to foreign antigens being continuously inhaled (Gollwitzer et al., 2014). However, recent discoveries have demonstrated that these localized microbial colonies may inadvertently create a pro-tumorigenic environment within the lungs (Segal et al., 2016). In a study in mice, Le Noci et al. (2018) demonstrated that the commensal lung microbiome could induce Foxp3^+^ T_regs_ that potentially increased growth in melanoma metastases in the lungs through immune suppression. However, aerosolized vancomycin or neomycin reduced lung microbiota resulting in downregulating the immunosuppressive IL-10 producing Foxp3^+^ T_regs_ populations. This resulted in a reduction of the metastatic nodule growth as well as an increase in T cells and natural killer T cell infiltration.

Tsay et al. (2018), using RNA-seq analysis of lower airway samples in lung cancer patients undergoing diagnostic bronchoscopy, found that translocation of taxa detected predominantly in the supraglottic region to the trachea was associated with upregulation of inflammatory pathways for p53 mutation, PI3K/PTEN, ERK and IL6/IL8. They concluded that enrichment of the lower airway with oral commensals may increase local immune tone with upregulation of IL1, IL6, and ERK/MARK, which in turn promotes tumor progression, thereby suggesting that microaspiration may be involved in the pathogenesis of lung cancer. In a study in human lung cancer patients undergoing tumor resection, Bailey et al. (2024) found that tracheal lavage specimens taken at the time of surgery contained a marked 16-, 6- and 6-fold higher abundance of the oral commensal species *Neisseria subflava*, *Granulicatella adiacens*, and the genus *Leptotrichia* in the lung cancer lavages versus controls, suggesting microaspiration and inflammation occur more significantly in lung cancer patients than in the control lavages.

### 4.3 Gut-lung axis microbiome connection

Despite their different anatomic locations, the gut and lung microbiomes are not isolated but rather entwined sharing molecular signaling pathways and microbial-immune crosstalk dubbed the gut-lung axis (Budden et al., 2017; Dang and Marsland, 2019). The direct microbial connection between these two anatomic areas likely comes from microaspiration of oral contents into the tracheobronchial tree. The gut-lung axis has been found to play a key role in various benign diseases including chronic inflammatory diseases, gastrointestinal disorders and most importantly chronic lung diseases such as asthma, allergy and chronic obstructive lung disease (Zhang et al., 2020). Particular attention has been focused on lung cancer with the gut and lung microbiotas’ modulating influence on the efficacy and response of lung cancer to immunotherapy and its toxicities (Hakozaki et al., 2020; Dora et al., 2023; Hu et al., 2024). Numerous clinical studies have shown correlations between the gut microbiota and the response of lung cancer patients to immunotherapy, although there are marked variations in actual taxa that lead to a positive treatment response (Dora et al., 2024). The differences likely are related to host genetics, diet and geographical location, and antibiotic use (Simpson et al., 2023). Studies are underway to employ various methods including probiotic supplements to modulate the gut microbiota to improve the patient response to immunotherapy (Zhao et al., 2025).

## 5 Lung microbiota

Being one of the largest organs by surface area and important for gas exchange, the lung represents a large interface between the external environment and its human host, and presents a unique site for host-microbiome interactions (Suau et al., 1999; Pilette et al., 2001). Through 16S rRNA sequencing, bacteria in both healthy lungs and diseased lungs have been identified as diverse microbial communities of distinct bacterial species (Harris et al., 2007; Charlson et al., 2011; Huang et al., 2011). Major phyla of the lung microbiome have been identified to belong to Bacteroidetes, Actinobacteria, Proteobacteria, and Firmicutes (Erb-Downward et al., 2011; Laroumagne et al., 2011). Major genera of the lung microbiome include *Pseudomonas*, *Staphylococcus, Corynebacterium, Streptococcus*, and *Neisseria* (Gomes et al., 2019). Based on these findings, the lung microbiota differ markedly from the gut or skin microbiome (Charlson et al., 2011), but instead share similarities with oral microbiota and the upper respiratory tract microbiome (Hilty et al., 2010).

In comparison to the gastrointestinal microbiome, the lung microbiome is surprisingly low in bacterial biomass with only an estimated 2,000 bacterial genomes per cm (Gilbert et al., 2018) surface area based on results of bronchial washings (Hilty et al., 2010; Mathieu et al., 2018). Low biomass is experimentally challenging during sequencing as most techniques for sequencing are designed for samples that contain a large biomass such as gut microbiome samples. Sequencing reads from low biomass specimens can result in erroneous frequencies and contamination in the 16S rRNA samples from the environment or from even the reagents used (Claassen-Weitz et al., 2020). Thus, experimentally-determined characteristics of the lung microbiome deserve particular consideration during analysis of clinical specimens and of published results.

### 5.1 Factors regulating the lung microbiome

Bacterial immigration, elimination, and replication (regional growth/reproduction rate) are the three major factors that regulate and maintain the composition of the lung microbiome and its abundance (**Table 4**) (Dickson et al., 2014; Dickson et al., 2015). Bacterial immigration can result from inhalation of air, consistently exposing the lung to air- or droplet-borne bacteria which can move bacteria from the upper respiratory tract into the lungs. Also, bacterial immigration commonly occurs from micro-aspiration of fluids from the oral cavity and these droplets seed the lungs. Elimination of bacterial microorganisms can be from coughing, mucous clearance, and innate or adaptive host immune responses (Dickson et al., 2014; Dickson et al., 2015). Regional Growth and Replication as well as Reproduction Rate depend on pH, temperature, oxygen tension, nutrient availability, local microbial competition, host epithelial cell interactions, activation of inflammatory cells, and concentration of inflammatory cells.

**Table 4:** Factors regulating the composition of the lung microbiome (Dickson et al., 2014, 2015).

Factors	Results
Bacterial immigration	Microaspiration, inhalation of bacteria and direct mucosal dispersion.
Bacterial elimination	Cough, mucociliary clearance, innate and adaptive host defenses.
Regional growth and replication	pH, temperature, oxygen tension, nutrient availability, local microbial competition, host epithelial cell interactions, activation of inflammatory cells and concentration of inflammatory cells.

**Figure 1:**
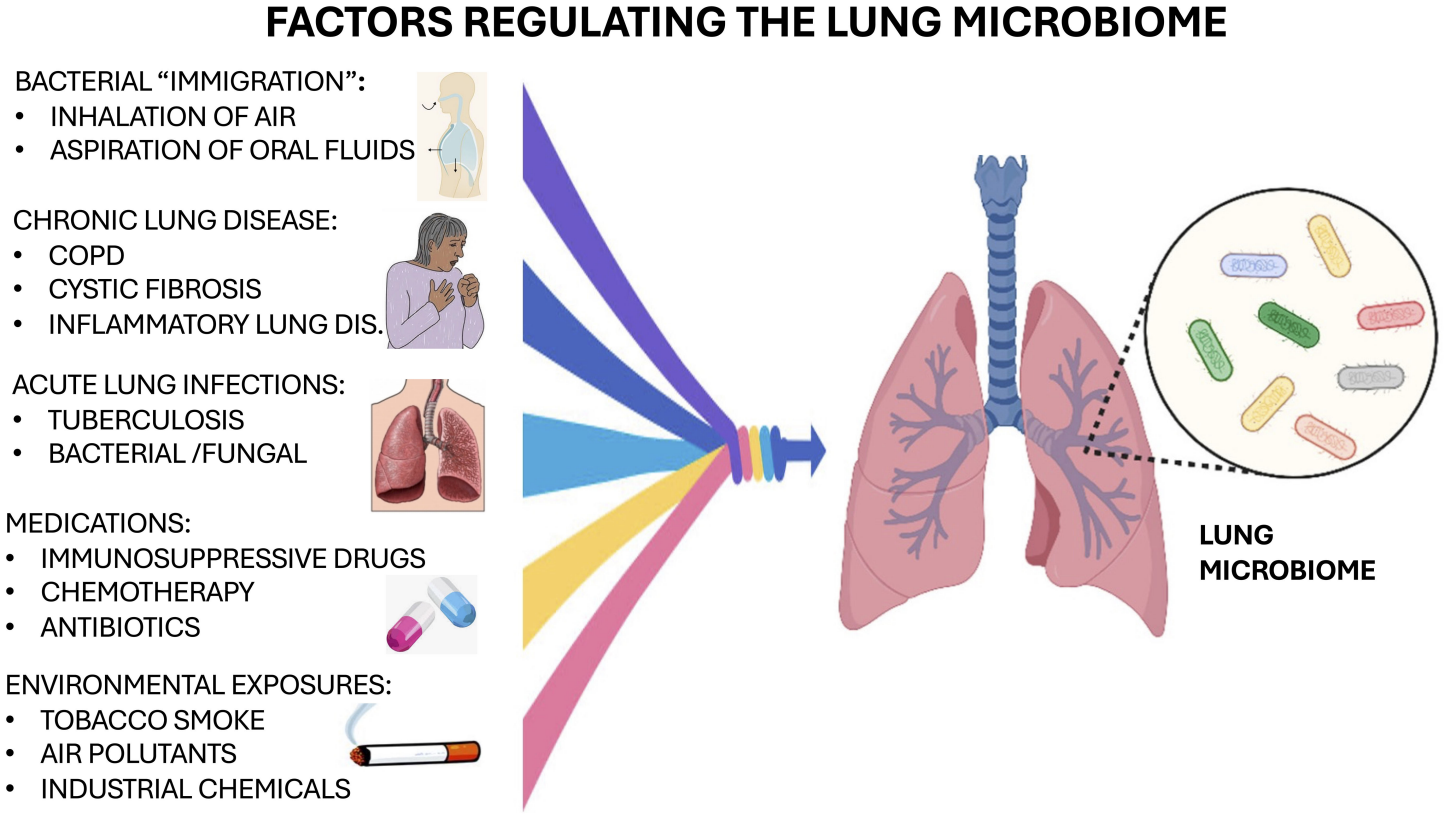
Factors regulating the lung microbiome.

The development of lung dysbiosis can occur rapidly by lung diseases causing a change in the local lung environment, as a result favoring the growth of specific bacterial microorganisms over others (Dickson et al., 2014). Substantial evidence indicates that local lung dysbiosis is linked to chronic pulmonary diseases often causing significant inflammation in the lungs (O’Dwyer et al., 2016). Patients with chronic obstructive pulmonary disease (COPD) had large microbial differences in their bacterial communities within the microenvironment and these were similar to lung samples from patients with cystic fibrosis (Sethi et al., 2007; Gomes et al., 2019). Recovery for these patients is difficult as continuous overgrowth of pathogenic organisms and new commensal strains in the lungs cause exacerbation of the disease (Akinosoglou et al., 2013). The composition of microbiota in the lungs is constantly changing depending on pathological conditions and can contribute to infection and recovery from specific lung diseases including cancer. Other important factors regulating the microbiome include the presence of chronic lung disease and acute infections, medications and environmental exposures, as illustrated in **Figure 1**.

### 5.2 Metabolic pathways modified by lung microbiota

Evidence suggests that lung microbiota may regulate pathways that are linked to oncogenic effects and can have a direct effect on driving tumor carcinogenesis. Dysbiosis may result in altered bacteria-derived molecules within the tumor microenvironment, giving rise to lung cancer cells with alterations in metabolic pathways and oncogenic signaling (Yu et al., 2016). These bacterial metabolites prove to be important for regulation of host metabolism and play a large role in signaling pathways (Yu et al., 2016). Providing further evidence, patients with lung cancer had a reported decreased abundance of the Kyoto Encyclopedia of Genes and Genomes (KEGG) database molecules within their lung microbiome, which are important molecules for energy metabolism and ATP-binding cassette (ABC) transport (Gustafson et al., 2010). In contrast, patients with lung cancer had an increase in molecules related to lipid metabolism, amino acid metabolism, and xenobiotic metabolism (Tsay et al., 2018). The dysbiosis shown in these metabolic profiles of lung microbiomes may impact gene expressions of epithelial cells within the airways.

*In vitro* studies conducted with samples of human lung adenocarcinoma, specifically with the A549 cell line, showed that bacterial products from this cell line resulted in upregulation of PI3K and ERK1/2 gene expression both of which are part of the signaling pathways. Transcriptomics of such cell lines and associated upregulated genes were consistent when lung cancer patients were compared to healthy individuals (Lloyd and Marsland, 2017). Interestingly, upregulation of the PI3K signaling pathway has been linked to early events that occur during promotion of lung tumor development (Tsay et al., 2018). These data, in combination with evidence from *in vitro* studies, suggest a direct association between the lung microbiome and tumorigenesis.

### 5.3 Immune microenvironment modified by microbiota

It has been suggested that microbiota may play a role in shaping the immune microenvironment which could potentially lead to a lung environment that promotes carcinogenesis. Many immune cells that are residents of the lung are important for maintaining homeostasis within pulmonary tissues and are providing immune surveillance against invading pathogens (Palucka and Coussens, 2016). Cancer development is associated closely with chronic inflammation promoted by inflammatory cells that secrete cytokines and chemokines. Another inflammatory cell product comprises prostaglandins which stimulate cell proliferation, tumor remodeling, and metastasis (Jin C. et al., 2019). Although these immune cells have been linked directly to inflammation, and evidence links inflammation to cancer promotion, the source of inflammation and how the immune system contributes to tumorigenesis cellularly and molecularly have yet to been discovered.

Jin Y. et al. (2019) collected lung adenocarcinoma samples that were driven by the KRAS point mutation and had a loss of function of the P53 gene to demonstrate how microorganisms could negatively impact inflammation and tumorigenesis in the lung. The authors found an association between lung tumorigenesis and increased bacterial density with an altered bacterial composition. The microorganisms within the adenocarcinoma samples were shown to stimulate products from myeloid cells such as Myd88-dependent IL-1β and IL-23, which resulted in the activation and the cell proliferation of certain residential lung cells. Such residential lung cells, particularly γδ T cells, began to give rise to immune cells that promote inflammation and neutrophil infiltration within the lung microenvironment, and led to upregulated IL-22 expression which has been shown to directly promote tumor cell proliferation (Fox and Wang, 2007). This study eliminated the commensal bacteria within mice through the administration of antibiotics or by creating germ-free mice. Antibiotic treatment was used to show that elimination of these microorganisms blocks the γδ T cells that downregulate the production of IL-17 which results in the suppression of lung tumor growth (Jin Y. et al., 2019).

### 5.4 Antibiotic modification of the microbiome

In a supporting clinical study, Le Noci et al. (2018) treated patients with an aerosolized combination of vancomycin and neomycin which resulted in a reduced number of lung tumor cell implantation. This patient study also showed that antibiotic treatment results in a decrease of IL-10 and an increase in natural killer cells and anti-tumoral T cells. Therefore, treating a patient with antibiotics may reduce immunosuppression by altering factors within the tumor microenvironment mediated by microbiota that would otherwise foster tumor growth.

Ongoing studies introduce the idea that certain bacterial compositions can contribute to upregulating lung inflammation and as a result contribute to lung cancer tumorigenesis. With antibiotic treatment, a shift in the tumor microenvironment, characterized by a decrease in gram-positive phylum *Firmicutes* and an increase in gram-negative phylum *Proteobacteria*, led to an upregulation of anti-tumor immunity (Le Noci et al., 2018). This emphasizes the significance of maintaining homeostasis within the lung microenvironment to promote the equilibrium of various bacterial species. Further evidence suggests that upregulation of oral microorganisms in the lung is associated with inflammatory responses. These oral taxa created increased levels of lymphocytes and expressed more inflammatory cytokines (Segal et al., 2016).

Gollwitzer et al. (2014) showed a shift from phyla *Firmicutes* to *Bacteroidetes* in neonates after birth and this shift in the lung tissues began to promote PD-L1 expression within lung dendritic cells. Although they did not specifically determine tumorigenic effects of bacterial shifts, it does suggest that changes in bacterial diversities can impact regulation of immune regulatory molecules, including PD-L1, and can also promote inflammatory factors within the innate and adaptive immune responses.

These diverse studies provided experimental insight to how the lung microbiome may contribute to lung carcinogenesis. More importantly, these studies present novel targets that could potentially be addressed to prevent cancers and even help with the treatment of lung cancers. Of note, while conducted rigorously, sample sizes were modest in these studies with significant variability in the results. Further characterization of the lung microbiome is needed to develop a bacterial biomarker for lung cancer. Also, comparison between lung cancer microbiomes to those of healthy subjects at a larger scale would need to be conducted to determine any significant differences between microbiomes and inform use of antimicrobial therapies in lung cancer patients.

## 6 Lung microbiota and carcinogenesis

**Figure 2:**
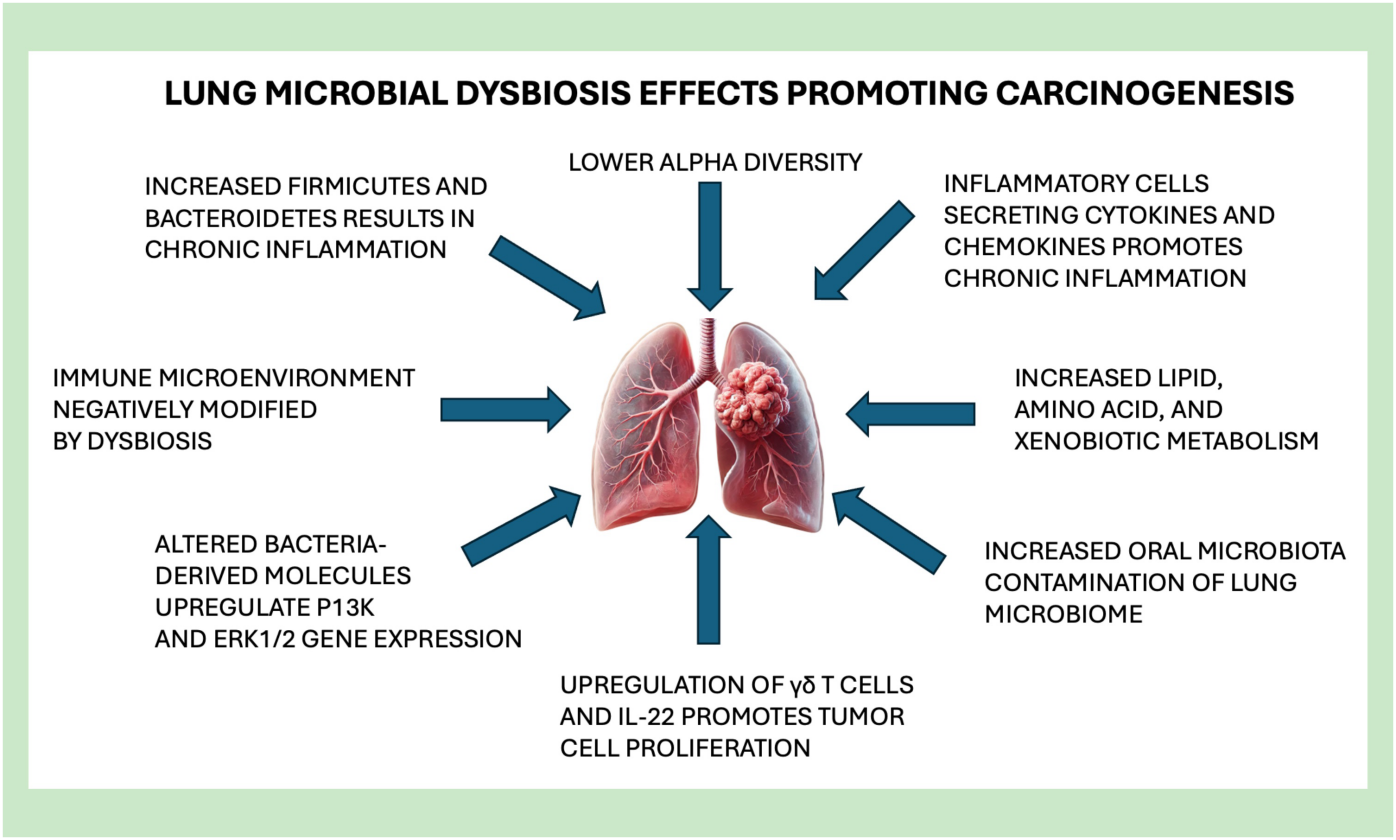
Lung microbial dysbiosis effects promoting carcinogenesis.

Lung cancer has been linked to microbial dysbiosis in many clinical studies with many possible mechanisms shown in **Figure 2**. Selected studies in **Table 5** illustrate these various mechanisms of how lung dysbiosis may promote lung carcinogenesis. Epidemiological studies have shown that patients with lung cancer have consistent bacterial infections in the lungs. Approximately 60% of patients with a diagnosed lung cancer have pulmonary infections that exacerbate the complications of cancer treatment, including post-obstructive pneumonia, negatively impacting the overall survival rate of patients with lung cancer (Yu et al., 2016). Antibiotic therapies are difficult to apply clinically due to the poor understanding of the microbiology in specific pneumonia infections. With recent discoveries, high throughput sequencing has contributed to elucidating the strong correlation of localized dysbiosis in the lung and the carcinogenesis of lung cancer. Microbiota of lung tumor samples were found to have a significantly lower alpha diversity, while bacterial compositions were related to the stage of cancer and epidemiologic exposures (Yan et al., 2015), in which the genus *Thermus* was significantly more abundant within the tumor tissue samples from patients with advanced staged disease. Also, the genus *Legionella* was significantly increased in patients who developed metastases, suggesting that certain microorganisms can affect cancer progression.

**Table 5:** Selected studies illustrating lung dysbiosis effects promoting carcinogenesis.

Study	Model	Pathway	Findings
Lloyd and Marsland, 2017	Lung cancer cell lines	Metabolic	Altered bacteria-derived molecules upregulate P13K and ERK1/2 gene expression
Tsay et al., 2018	Humans with transbronchoscopic specimens	Metabolic	Increase in molecules related to lipid metabolism, amino acid metabolism, and xenobiotic metabolism
Jin Y. et al., 2019 Fox and Wang, 2007	Humans with adenocarcinoma	Immune microenvironment promoting inflammation	Immune environment modified by microbiota that promote inflammation and neutrophil infiltration within the lung microenvironment, and led to upregulated IL-22 expression which directly promoted tumor cell proliferation
Tsay et al., 2018 Segal et al., 2016 Bailey et al., 2024	Humans	Lung inflammation with dysbiotic microbiome	Enrichment of the lower airway with oral commensals by microaspiration may increase local immune tone with upregulation of IL1, IL6, and ERK/MARK, which in turn promotes tumor progression
Laroumagne et al., 2011 Liu et al., 2018	Humans	Diversity	Alpha diversity (species diversity within a specific sample) was significantly lower in lung cancer tumor tissue when compared to non-malignant lung tissues
Hosgood et al., 2014	Humans	Bacterial phyla	Firmicutes and Bacteroidetes were present at a significantly higher ratio in the smoking group with lung cancer compared to the non-smoking group
Jin C. et al., 2019 Pathria et al., 2019	Germ-free mice and humans	Inflammatory cells	Tumor associated macrophages produce cytokines and chemokines within the tumor environment that can suppress cytotoxic T cells, thus helping to promote tumor growth and even foster tumor metastasis

Other taxa of bacteria also have been shown to have high association with lung cancers through various studies. Microorganisms such as the genera *Veillonella* and *Capnocytophaga* were increased in saliva samples from patients with lung cancer and could possibly be used as an early detection biomarker for patients with suspected adenocarcinoma and small cell carcinomas (Lee et al., 2016). Increased abundance of the genera *Megasphaera* and *Veillonella* from bronchoalveolar lavage samples were detected by Bailey et al. (2024) from patients with lung cancer (Bailey et al., 2024), while Cameron et al. (2017) discovered seven opportunistic pathogens in sputum samples from lung cancer patients. Other studies showed a correlation of other microorganisms and specific lung cancers, and they identified enriched taxa in patients who were chronic smokers (Greathouse et al., 2018). Importantly, there are *significant* variations in the actual organisms found in these diverse studies, as there has been in many other microbiome studies.

Lung cancer is commonly associated with dysbiosis and chronic inflammation in the local lung microbiome measured by bacterial abundance, alpha and beta diversities, and alterations in bacterial composition. While bacterial taxa remained diverse in comparison with various study findings, it is important to note that the sampling method, sampling type, lung cancer diagnosis, and patient cohort contributed to variability between the studies. Nonetheless, each study was able to demonstrate a correlation between microorganisms and lung cancer detection or carcinogenesis. Although the specifics of bacterial dysbiosis in lung cancers have not been determined, most importantly a majority of these studies have shown that increased alpha diversity seems to correlate with better treatment responses and overall survival (Nicholson et al., 2012). These recent advances have created investigator interest in finding a diagnostic biomarker for lung cancer detection and potentially metastasis, with many studies ongoing.

### 6.1 Lung microbiota in lung cancer

Epidemiologic studies have discovered many relationships between the microbiome and lung cancer, a malignancy with the highest cancer-related death rate worldwide. For example, studies found significant relevance of the species *Mycobacterium tuberculosis* in early lung cancer studies (Liang et al., 2009). The link of *M. tuberculosis* infection to lung cancer has been suggested to be due to chronic inflammation-associated carcinogenesis (Christopoulos et al., 2014). Also, persistent infection of *M. tuberculosis* increases production of tumor necrosis factor (TNF) resulting in chronic pulmonary inflammation. Chronic pulmonary inflammation eventually leads to pulmonary fibrosis which is also linked to the development of lung cancers. In turn, people with lung cancer also have an increased risk of *M. tuberculosis* reactivation infections as they become immunocompromised during and after their chemotherapy treatments (Morgan and Huttenhower, 2012). As *M. tuberculosis* is now predominantly present in lower-income countries, its association with lung cancer is less prominent in higher income countries.

Other epidemiologic studies have provided increased evidence that the changes of the microbiome may be significant contributing factors to the development of lung cancer. Between non-malignant and tumor tissues, beta diversity (differences in species composition between different samples) was not significantly different (Yu et al., 2016). However, alpha diversities (species diversity within a specific sample) were significantly lower in lung cancer tumor tissue when compared to non-malignant lung tissues (Laroumagne et al., 2011). Several microorganisms have been analyzed to show their effects in the development and progression in cancer cases when compared to control cases. Bronchoscopic samples from a study by Laroumagne et al. (2011) of 388 cases helped the investigators identify pathogenic gram-negative bacteria that colonized the bronchi in 40% of lung cancer patients including species *Escherichia coli*, *Haemophilus influenzae*, and *Enterobacter* spp (Hosgood et al., 2014). Streptococcus, Granulicatella, and Abiotrophia genera were more abundant in oral and sputum samples from women in China with lung cancer when compared to healthy controls (Yan et al., 2015). In the same study, the alpha diversity in sputum samples from lung microbiota was higher in cases when women used smokey coal for heating and cooking compared to women who used smokeless coal. Interestingly, those same subjects did not show any significant contrast in alpha diversity in the oral samples (Yan et al., 2015).

Lee et al. (2016) looked at the microbiome of bronchoalveolar lavage fluid in squamous cell carcinoma (SCC) and adenocarcinoma patients compared to benign nodules and discovered that there were significant variations in the presence of the organisms. The genera Capnocytophaga, Selenomonas, Veillonella, and Neisseria were found in malignant samples when compared to the benign mass-like lesion controls. Because of these findings, they posited that lung cancer screenings for bacterial biomarkers of genera Capnocytophaga and Veillonella may show promise in the prediction of SCC and adenocarcinoma. In that pilot study, Lee et al. (2016) discovered that phyla Firmicutes and Saccharibacteria (TM7), and genera Veillonella and Megasphaera in bronchoalveolar lavage fluid were relatively more abundant from patients with lung cancer. Of the patients with lung cancer, the phyla Firmicutes and Bacteroidetes were present at a significantly higher ratio in the smoking group compared to the non-smoking group (Man et al., 2017).

Phylum TM7 was notably higher in lung cancer and COPD cases which suggests that COPD may contribute to development of lung cancer possibly from chronic inflammation. With this discovery, Man et al. (2017) suggested a potential biomarker for lung cancer would have significantly high AUC values due to the combination of increased genera Megasphaera and Veillonella (Lee et al., 2016).

Lung microbiome research is typically done by analyzing saliva, sputum, and trans-bronchoscopic bronchoalveolar lavage fluids samples, since lung biopsies are obviously not practical for healthy subjects. This makes categorizing the normal lung microbiome more difficult as these samples can be easily contaminated by the upper respiratory tract, and more accurate analysis and assessments of the lung microbiome would need to come from lung tissues (Yu et al., 2016). Researchers also discovered that the lung microbiota was different when compared to the digestive tract microbiota within healthy subjects (Liu et al., 2018). In lung tumor tissue, lower alpha diversity was observed compared to normal lung tissue which is analogous to results observed when comparing other respiratory diseases to healthy lung tissues.

Liu et al. (2018) compared the lung microbiome of SCC and adenocarcinomas and observed decreased abundance of the genus Ralstonia and increased abundance of the genus Thermus in adenocarcinoma compared to SCC. They suggested that microbiota composition and abundance could be associated with cancer histology. The same study further revealed that in metastasis, the genus Legionella was abundantly higher suggesting that tumor progression may be mediated through this microorganism (Liu et al., 2018). They reported that patients with lung cancer had a significant decrease in their lung microbial diversity in comparison to the controls. Also, they observed a steady decline of alpha diversity from the healthy noncancerous site compared to the cancerous site by testing healthy controls that underwent a bronchoscopy versus lung cancer patients with unilateral lobar masses (Walters et al., 2013). In control samples without cancer, the genus Staphylococcus was more abundant, while the genus Streptococcus was more abundant in samples from patients with cancer. This suggests that the development of lung cancer could correlate with microenvironmental changes (Walters et al., 2013), although there are significant differences in the actual genera that are most important, which is a common weakness of these studies.

## 7 Lung cancer treatment and the microbiome

Diagnosed lung cancers require various treatment approaches depending on stage, using surgical resection, radiation therapy, chemotherapy and/or immunotherapy. Unfortunately, lung cancer has a high rate of late-stage diagnosis with 75% of patients being diagnosed in an advanced stage III or IV (Walters et al., 2013). Despite the many advances in lung cancer treatment, options for late-stage disease are often limited. Due to the staggeringly high late-stage diagnosis rate, it has become increasingly urgent to discover early-stage detection and treatment techniques to improve the prognosis for these patients.

### 7.1 Microbial effects for diagnosis and treatment in lung cancer

**Figure 3:**
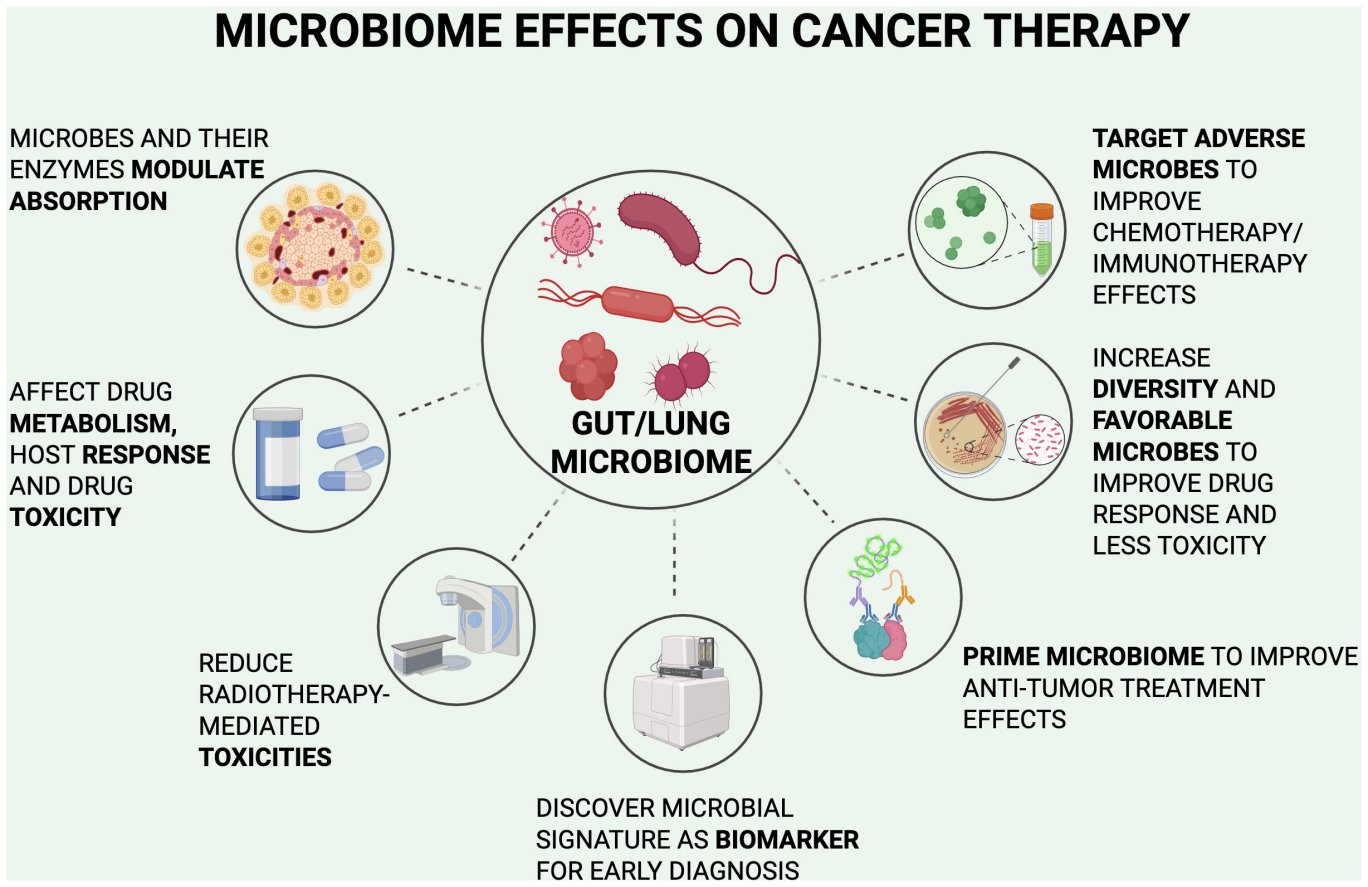
Microbiome effects on cancer therapy.

Currently, our understanding of the relationship between gut microbiota and lung cancer is primitive, with many treatment studies limited to *in vitro* and non-clinical approaches. The close bidirectional interaction of the gut-lung axis suggests why interventions for the gut microbiome result in significant effects in the lungs and its malignancies. Clinical studies have included the increased use of probiotics, anti-inflammatory diets, and more invasive options including fecal microbiota transplants (FMT). With the understanding of the relationship between the gut microbiota and lung cancer, an approach for new early-stage detection methods may increase the number of treatment options available for lung cancer and improve outcomes for patients managed with current treatment options. **Figure 3** summarizes the following microbiome effects.

### 7.2 Microbiome targets

Products that target the microbiome have been commercially available for decades, such as probiotic and prebiotic supplements, and recent advances in microbiome research have suggested potential clinical benefit and safety of these products. Clinical data from the use of probiotics and prebiotics suggest improved integrity of the gastrointestinal tract while promoting microbial homeostasis, metabolism regulation through vitamins and short-chain fatty acids, and even neutralization inflammatory agents and carcinogens. Hemarajata and Versalovic (2013) concluded that maintaining diversity and relative concentrations of the phyla Bacteroidetes, Actinobacteria, Proteobacteria, and others in the gut microbiome through the administration of probiotics and prebiotics promotes optimal homeostasis of the host immune system.

Based on this information, researchers have investigated potential microbial drug targets as therapies for lung cancer. Microbial drug targets have the potential to be addressed by targeted therapies, thereby decreasing adverse events that are a result of chemotherapy treatment.

A clinical trial by de Jong et al. (2006) suggested that irinotecan treatment of small cell lung cancer with concurrent administration of neomycin was of clinical benefit in decreasing the adverse events caused by the chemotherapy drug. A preclinical study treating mice with colon cancer by inhibiting a bacterial enzyme, β-glucuronidase, also alleviated chemotherapy drug toxicity by protecting the mice from irinotecan-mediated diarrhea (Wallace et al., 2010).

Despite such medical breakthroughs, the current gaps in knowledge and lack of investigational support to study how beneficial microorganisms and their molecular mechanisms can benefit the host prevent researchers from understanding how the microbiome interacts with its host. It is unknown whether changes in the microbiome will result in imbalance of homeostasis and cause disorders, or even cause inflammatory responses that could result in the formation of precancerous lesions. Also, FDA recently issued a safety alert regarding fecal microbiota transplantation (FMT) due to potential risks of serious or life-threatening infections resulting from pathogenic organisms being disseminated from the original donor (FDA, 2020).

### 7.3 Radiation therapy and the microbiome

Radiation therapy for early and advanced stage lung cancer, despite its many side effects, has been widely accepted as routine treatment in clinical practice. Unfortunately, radiotherapy can result in unexpected adverse events due to radiation toxicities and damage the host immune system. Studies of the effects of radiotherapy on the gut microbiome remain scarce and research in this field has not progressed significantly. Cui et al. (2017) had a particular interest in the relationship of gut microbiota and radiation-induced side effects. In their preclinical study of mice receiving a FMT, results showed a decrease in radiation damage that did not promote tumor cell proliferation *in vivo*. However, Gerassy-Vainberg et al. (2018) showed radiation-induced proinflammatory dysbiosis with enhanced TNF-α, IL-1β, and IL-6 within microbial signature expressions in post-radiated mice when compared to microbiota in radiation naïve mice. These studies are important in predicting microorganisms that are hypersensitive to radiation. By identifying these microorganisms, they can then be targeted to improve curative radiation effects. Although such studies are very preliminary, in the future microbiota may be able to serve as a premise for therapeutic strategies to help reduce radiotherapy-mediated toxicities and even to improve the prognosis of lung cancer patients post-radiation treatment (Raza et al., 2019).

### 7.4 Gut microbiome and drug metabolism

Recent studies have implied that the gut microbiome is vital to drug metabolism, host response sensitivity, and chemotherapy toxicities (Roy and Trinchieri, 2017). Microorganisms and microbial enzymes within the gut microbiota have proven to directly modulate the host response to drug absorption and metabolism (Montassier et al., 2015). Gut microbiota can additionally regulate gene expression thereby indirectly affecting the rate of drug metabolism both with oral and IV administrations, as well as modulating the mucosal barrier response and changing the physiology of distant organs (Selwyn et al., 2016). *In vivo* and *in vitro* experiments demonstrated a complex relationship between human microorganisms and chemotherapy drugs. Of note, certain microbial species are able to promote the alkylating agent CB1954 in blood circulation and inhibit gemcitabine demonstrating that local bacteria can in fact change the efficacy of chemotherapeutic agents (Lehouritis et al., 2015).

Genotoxic platinum drugs are another class of antineoplastic therapeutics that contribute to anti-cancer treatment outcomes by inhibiting DNA replication (Wagner and Karnitz, 2009). This subset of drugs targets the plasma membrane and mitochondria of tumor cells, but are destructive to the host as well, leading to serious side effects including deafness, and compromising the blood-brain barrier’s integrity (Abuzeid et al., 2009). A contributing factor to the side effects of genotoxic platinum drugs may derive from the destruction of the mucosal layer which results in pathogenic microorganisms’ invasion of mesenteric lymph nodes and possibly entry into the bloodstream as well (Hooper and Macpherson, 2010). Additionally, a number of studies have shown that antibiotic misuse may not only aggravate the side effects from the administration of antineoplastic drugs, but they may potentially cause severe systemic adverse events (Zitvogel et al., 2018).

Most studies of the microbiome and chemotherapy drug administration remain within animal models and only a few trials have been conducted in humans to explore the alterations of the gut microbiome and its functions during chemotherapeutic treatment and post-chemotherapy surveillance of patients being treated for lung cancer. Moreover, large clinical trials are required to investigate if modulation of gut microorganisms will aid in the treatment of lung cancer during chemotherapy and determine if those microorganisms contribute to minimizing drug toxicities.

### 7.5 Priming the gut microbiome

Several potentially effective ways to prime the gut microbiome may serve to influence outcomes for patients treated with immunotherapies (**Table 6**). Some studies have contributed to the hypothesis that dysbiosis within the intestinal microbiota may also affect the outcomes of immunotherapies for patients with cancer (Routy et al., 2018). For example, a study in France by Routy et al. (2018) was conducted with 249 lung cancer patients receiving immunotherapy to target a PD-1 mutation. Of the 249 patients, 69 were treated with antibiotics because they had underlying diseases with onset before the first cycle of treatment which disrupted their intestinal microbiome. Upon follow up, it was determined that the 69 antibiotic-treated patients had shorter progression-free survival and overall survival compared to patients that did not receive antibiotics during immunotherapy treatment. These findings suggest that antibiotic use could negatively impact the efficacy of immunotherapy treatment and reduce the effectiveness of the drug (Routy et al., 2018). Nyein et al. (2022) also found that antibiotics administered prior to or during immunotherapy or chemotherapy treatment of advanced lung cancer resulted in a worse response rate and worse overall survival, likely resulting from a disruption of the eubiotic gut microbiome.

**Table 6:** Ways to prime the gut microbiome.

Factors	Effects
Avoiding antibiotics (Routy et al., 2018; Nyein et al., 2022)	Avoid antibiotics since they negatively impact the efficacy of immunotherapy treatment and reduce the effectiveness of the drugs
Anti-inflammatory diets (Gill et al., 2022)	Promotes the growth of beneficial bacteria and enhancing their anti-inflammatory functions in the gut and throughout the body
High fiber diets (Fu et al., 2022)	Promotes a healthy microbiome by providing food for beneficial gut bacteria, which helps them thrive and maintain a balanced ecosystem. Fiber is fermented by gut bacteria produces short chain fatty acids like butyrate
Administration of probiotics to increase microbial diversity (Jin Y. et al., 2019)	Higher microbial diversity, which is considered a favorable microbiome, exhibited an enhanced microbial signature specifically for memory T cells and natural killer cells in their blood samples
Prebiotics, probiotics and postbiotics (Gopalakrishnan et al., 2018; Li and McAllister, 2022)	Results in higher diversity of the microbial community, which positively correlates with cancer cells to being targeted and killed more effectively due to increased T cell activity
Fecal microbiota transplants ± probiotics (Gopalakrishnan et al., 2018; Li and McAllister, 2022)	Higher diversity of the microbial community positively correlates with cancer cells to being targeted and killed more effectively due to increased T cell activity
Limiting or increasing certain microbiota (Shi et al., 2020; Li and McAllister, 2022; Yue et al., 2022)	*Akkermansia muciniphila* and other specific probiotic organisms have demonstrated positive contributions to cancer immunotherapy
Prime TRL4-signaling (Paulos et al., 2007)	Commensal bacteria in the host gut could potentially activate myeloid cells associated with a tumor
Exercise (Varghese et al., 2024)	Physical activity promotes beneficial bacteria, improves absorption of nutrients, and supports a balanced gut microbiome, resulting in better immune and metabolic functions

The species *Akkermansia muciniphila* is a probiotic organism that has been shown in recent studies to prevent obesity and diabetes, but has also demonstrated positive contributions to cancer immunotherapy (Li and McAllister, 2022). A recent review described a comparison of the gut microbiota for two groups of patients after isolating *Akkermansia muciniphila* from their stool samples. The investigators then performed a FMT into germ free mice and treated them with PD-1 inhibitors. They found that mice receiving feces from a probiotic-treated patient were able to respond rapidly to the PD-1 inhibitors when compared to mice that received a FMT from a patient without the probiotic treatment. Additionally, the researchers determined that immunotherapy response can be restored by using oral *Akkermansia muciniphila* probiotics as well (Gopalakrishnan et al., 2018; Li and McAllister, 2022). This outcome could be explained by the higher diversity of the microbial community, which positively correlates with cancer cells to being targeted and killed more effectively due to increased T cell activity. In contrast, increased regulatory T-cell activity within a host with unfavorable bacteria may suppress the immune response in that patient.

Jin Y. et al. (2019) conducted a clinical study in China focusing on patients diagnosed with advanced stage non-small cell lung cancer and treated with immunotherapy targeting the PD-1 checkpoint inhibitor. Patients with greater diversity in their gut microbiota responded better to the anti-PD-1 immunotherapy checkpoint inhibitors. Patients with higher microbial diversity, which is considered a favorable microbiome, exhibited an enhanced microbial signature specifically for memory T cells and natural killer cells in their blood samples. Similarly, the systemic administration of a certain bacterium from the genus *Bifidobacterium*, can stimulate signaling in the cyclic GMP-AMP synthase (cGAS)-stimulator of interferon genes (STING) pathway and increase the performance of dendritic cells after anti-PD-1 treatment. *Bifidobacterium* administration successfully converted mice that were non-responders into mice that responded to immunotherapy (Shi et al., 2020).

Most recently Lin et al. (2025) reported that they isolated a new strain of the bacteria genus Hominenteromicribium from the feces of 50 patients who responded to PD-1 blockade therapies. This strain *Hominenteromicrobium Mulieris* YB328 was instilled into mice treated with anti-PD-1 had higher abundances of activated CD8^+^ T cells and cytokine-producing CD8^+^ T cells in the tumor microenvironment than mice treated with the common commensal species *Phocaeicola vulgatus* (found in non-responder feces) and anti-PD-1 or control mice. YB328 administration alone did not inhibit tumor growth. However, “patients with elevated YB328 abundance had increased infiltration of CD103^+^CD11b^−^ cDCs in tumours and had a favourable response to PD-1 blockade therapy in various cancer types.” They also analyzed “whether the bacterial species *Akkermansia muciniphila* and *Bifidobacterium longum*, which reportedly improve responses to cancer immunotherapy, possessed properties similar to those of YB328.” *A.* *muciniphila*, but not *B.* *longum*, demonstrated a marked anti-tumor effect when combined with anti-PD-1 treatment (Lin et al., 2025). Further studies are needed to discern whether any particular bacterial genus or species will provide reproducible benefit if given prospectively as a microbial “adjuvant” in humans. Nevertheless, numerous preclinical and early clinical studies have identified bacterial taxa including Ruminococcaceae, Akkermansia, and Bifidobacterium that are associated with improved response to immune checkpoint inhibitors. Numerous microbiome-focused interventions are undergoing clinical evaluation, although the field is in early stages now and will require large-scale trials before microbiome interventions enter routine clinical practice (Barragan-Carrillo et al., 2025).

The gut microbiome can also be primed to support an immune response via TLR4-signaling (Paulos et al., 2007). Commensal bacteria in the host gut could potentially activate myeloid cells associated with a tumor. This would result in a production of inflammatory cytokines and tumor-necrosis factor (TNF) that help modulate the tumor microenvironment and the anti-tumor effect during immunotherapeutic treatments (Iida et al., 2013). Each of these studies helped reveal the close relationship between gut flora and cancer immunotherapeutic treatment outcomes which informs potentially improved efficacy for immunotherapies. Understanding the positive and negative associations of the intestinal microbiome with cancer immunotherapy can inform study designs that explore cancer treatment outcomes by limiting or increasing certain microbiota. Nevertheless, understanding the relationship between the gut microbiome and cancer treatments needs to be explored in a microbe specific manner and in larger studies.

### 7.6 Diagnostic tools

Lung cancer is typically found through chest x-rays or by screening low-dose chest CT scans. Risk stratification factors that group patients into low-risk and high-risk include age, gender, smoking history, and occupational or environmental exposures. Although these guidelines for screening high-risk individuals have been recommended for the last decade (U.S. Preventive Services, 2021), many patients who develop lung cancer fall outside of these demographics. For that reason, finding specific high-risk microorganisms, microbial signatures, or microbial alterations that are associated with lung cancer would provide a better risk stratification for high- or low-risk patients, thus improving the high-risk screening population. As sequencing capabilities expand, comparison of microbiomes between different patients, exposures, and multiple diseases have piqued the interest of researchers and physicians. It has been documented that microbial flora alterations and the development of lung cancer display significant correlation (Hosgood et al., 2014), as shown by various epidemiological studies with long-term observations and where relevant samples were provided (Lee et al., 2016; Chalmers et al., 2018; Liu et al., 2018).

Prior studies have demonstrated that alterations in the microbiome have been linked to the development and exacerbation of various lung diseases. These diseases are characterized by high inflammatory responses and have the potential to progress to lung cancer (Garcia-Nuñez et al., 2014; Taylor et al., 2017; Zheng et al., 2020). Zheng et al. (2020) discovered microbial signatures within the gut microbiome that predicted early-stage detection in lung cancer. Similarly, Yan et al. (2015) observed that concentrations of the genera *Neisseria, Streptococcus* and *Porphyromonas* in saliva from lung cancer patients were significantly higher than those in samples provided by patients without lung cancer, potentially serving as a biomarker for early-stage disease detection. Pilot studies using 16S rRNA sequencing have determined greater abundance of certain microorganisms including the families Bacteriodaceae, Lachnospiraceae, and Ruminococcaceae in lung tissues that correlate with a decreased rate of recurrence-free survival and disease-free survival rates (Peters et al., 2019). The importance of conducting additional clinical studies to establish sensitive and specific microbial biomarkers for lung cancer is a desirable focus. Such research will determine whether microbial biomarker tests can be developed that meet clinically valid criteria for sensitivity, reliability, and reproducibility.

## 8 Conclusion

Advances in microbiome technologies have enhanced our understanding of the complex interplay between microbial communities and cancer, particularly in tumor development, immune system interactions, treatment outcomes, and survival. Cancer immunoediting explains how tumors may evade immune surveillance. Whereas, targeting immune-related molecular pathways holds promise to reduce immunoresistance and inhibit tumor growth. These insights have also driven the development of therapies that boost anti-tumor immune responses, showing encouraging clinical results.

Chronic inflammation, often linked to persistent infections, plays a critical role in cancer pathogenesis. Although the relationship between microbiota and inflammation requires further study, it is well recognized that microbial-induced inflammation promotes tumor growth. Chronic diseases like chronic obstructive pulmonary disease increase lung cancer risk by fostering inflammation and persistent infections, which exacerbate other comorbidities and impair survival during cancer treatment. Some microbial species contribute to cancer progression by suppressing immune responses and evading immunosurveillance.

The lungs’ direct exposure to the external environment makes them vulnerable to inflammatory and tumorigenic microbes. Migration of oral microbiota into the tracheobronchial tree by microaspiration adds to the adverse organisms, further contributing to an inflammatory, dysbiotic lung microbiome. Despite the occasional inconsistencies in the published studies with its somewhat contradictory evidence, recent studies of the lung microbiome have identified localized microbial communities that may contribute to excessive inflammation and carcinogenesis. Some microbes also promote metastatic tumor growth, as seen in lung metastases from melanoma.

Technological progress in deep sequencing, especially 16S rRNA analysis, has been instrumental in identifying microbial signatures consistently present in lung cancers. While no single microbial species or signature currently serves as a reliable lung cancer biomarker, higher microbial alpha diversity correlates with improved treatment responses and lower recurrence risk. The gut microbiome also influences lung cancer through the gut-lung axis, with this bidirectional communication amplifying systemic inflammation.

Although the lung microbiome has lower biomass than other organs, studies show strong associations between specific genera and lung cancer presence. Microbial communities in both the lung and gut influence treatment outcomes. Probiotic use during cancer therapy appears to support microbial homeostasis and maintain diversity despite immune suppression from chemotherapy and radiation, leading to better outcomes. Microbial diversity also enhances chemotherapy and immunotherapy efficacy by boosting immune responses.

The ongoing pursuit of microbial biomarkers is promising, with advancements in sequencing technologies likely to identify species or microbial signatures critical for lung cancer diagnosis and personalized treatment strategies. Continued research is essential to translate these microbial insights into clinical applications.
